# A pooled testing system to rapidly identify cattle carrying the elite controller BoLA‐DRB3*009:02 haplotype against bovine leukemia virus infection

**DOI:** 10.1111/tan.14502

**Published:** 2021-12-19

**Authors:** Kosuke Notsu, Hala El Daous, Shuya Mitoma, Junzo Norimine, Satoshi Sekiguchi

**Affiliations:** ^1^ Graduate School of Medicine and Veterinary Medicine University of Miyazaki Miyazaki Japan; ^2^ Faculty of Veterinary Medicine Benha University Toukh Egypt; ^3^ Department of Veterinary Science, Faculty of Agriculture University of Miyazaki Miyazaki Japan; ^4^ Center for Animal Disease Control University of Miyazaki Miyazaki Japan

**Keywords:** allele‐specific PCR, BoLA‐DRB3*009:02, bovine leukemia virus, elite controllers, pooled samples, TaqMan assay

## Abstract

As genetically resistant individuals, the “elite controllers” (ECs) of human immunodeficiency virus infection have been focused on as the keys to developing further functional treatments in medicine. In the livestock production field, identifying the ECs of bovine leukemia virus (BLV) infection in cattle is desired to stop BLV transmission chains on farms. Cattle carrying the bovine leukocyte antigen (*BoLA*)‐DRB3*009:02 allele (DRB3*009:02) have a strong possibility of being BLV ECs. Most of cattle carrying this allele maintain undetectable BLV proviral loads and do not shed virus even when infected. BLV ECs can act as transmission barriers when placed between uninfected and infected cattle in a barn. To identify cattle carrying DRB3*009:02 in large populations more easily, we developed a pooled testing system. It employs a highly sensitive, specific real‐time PCR assay and TaqMan MGB probes (DRB3*009:02‐TaqMan assay). Using this system, we determined the percentage of DRB3*009:02‐carrying cattle on Kyushu Island, Japan. Our pooled testing system detected cattle carrying the DRB3*009:02 allele from a DNA pool containing one DRB3*009:02‐positive animal and 29 cattle with other alleles. Its capacity is sufficient for herd‐level screening for DRB3*009:02‐carrying cattle. The DRB3*009:02‐TaqMan assay showed high‐discriminative sensitivity and specificity toward DRB3*009:02, making it suitable for identifying DRB3*009:02‐carrying cattle in post‐screening tests on individuals. We determined that the percentage of DRB3*009:02‐carrying cattle in Kyushu Island was 10.56%. With its ease of use and reliable detection, this new method strengthens the laboratory typing for DRB3*009:02‐carrying cattle. Thus, our findings support the use of BLV ECs in the field.

## 1 INTRODUCTION

Retroviruses infect a wide range of mammals and cause various disease processes in them including immunodeficiencies, neurological disorders, and tumor development.^1^ The pathogenicity is heterogenous and depends on the virus–host interaction. Interestingly, there are rare patients with the in‐built ability to keep human immunodeficiency virus (HIV) proviral loads below the detection limits of viral load assays without receiving antiviral therapy, the so‐called “elite controllers” (ECs).^2^ They are understood to have specific HLA haplotypes (B*27, B*57, and B*14 alleles).^3^, ^4^, ^5^ Although attention has been directed at ECs as potential keys to opening further functional treatments in medicine, the percentage of ECs in the population is less than 1%.^6^ The desirability of identifying the ECs of retroviruses also exists in the livestock production field. Therefore, identifying them would help to stop the spread of bovine leukemia virus (BLV), for which no vaccines or treatments are available. To identify these seldomly observed ECs in animals requires the development of a high‐throughput genotyping identification strategy.

BLV, a *Deltaretrovirus* genus member within the *Retroviridae* family, causes the malignant B‐cell lymphoma known as enzootic bovine leukosis. The similar structure and properties of BLV make it closely related to human T‐lymphotropic virus type 1. BLV transmits via infected lymphocytes from infected cattle to uninfected ones. Infected cattle remain infected over their lifetimes. Upon infection with BLV, 30% of cattle develop persistent B‐cell lymphocytosis, and fewer than 5% develop lymphosarcoma after a long latent period.^7^, ^8^, ^9^ BLV infection, a chronic wasting disease, is responsible for reduced milk productivity and fertility rate decline, and is a lifelong illness even when the disease caused by it (enzootic bovine leukosis) becomes subclinical.^10^, ^11^, ^12^, ^13^

The high global prevalence of BLV makes its eradication in the field by culling all the cattle infected with it economically impractical.^14^ Therefore, keeping BLV‐infected cattle requires the establishment of a BLV control strategy. The current BLV control strategies used for within‐farm transmission are based on isolating BLV‐infected cattle from the herd or barn.^15^ They also involve avoiding other iatrogenic factors such as the repeated use of contaminated needles, dehorning, and rectal palpation via the use of a common sleeve.^16^, ^17^, ^18^ Implementing these control strategies, however, requires enough space to separate the animals and this can be costly. Thus, effective BLV control is limited by a farm's size capacity.

Another control option for BLV involves the use of BLV ECs, which focuses on halting the within‐herd BLV transmission chain.^12^ Haplotypes of the bovine leukocyte antigen (*BoLA*) *class II‐DRB3* exon 2 region are associated with disease susceptibility in individuals.^19^ Cattle carrying the BoLA class II‐DRB3*009:02 allele (DRB3*009:02) are known to be strongly associated with BLV dissemination resistance. Most of cattle carrying this allele results in an undetectable provirus status and arrested disease progression, even when infected.^20^, ^21^, ^22^, ^23^, ^24^ A previous field study reported that cattle carrying DRB3*009:02 with absence of provirus were not the transmission sources of BLV.^23^ Based on these knowledge, property of carrying DRB3*009:02 is useful marker for the screening of BLV ECs. On the other hands, minor population of cattle carrying DRB3*009:02 have been reported to have detectable BLV provirus and progress lymphoma^25^, ^26^, ^27^, ^28^ because BLV resistance seems to be determined by the combination of *DRB3* heterozygous alleles.^29^, ^30^ It is important to confirm not only DRB3*009:02 but also undetectable provirus level in BLV infected cattle. BLV ECs could be used for BLV transmission control by using them as barriers between BLV‐infected and uninfected cattle, even when the farmer lacks enough space to separate them. However, at less than 10% of a population, cattle carrying this allele are rare.^24^ Hence, genotyping all cattle to identify the relevant allele in a large population has huge costs and is laborious. To tackle this problem, pooled testing is a potentially useful screening method for identifying the rare target animals in a large population because of its time and cost advantages. Indeed, it has been used for surveying, monitoring, and epidemiological disease investigations.^31^, ^32^, ^33^, ^34^ Therefore, the objective of this study was to develop a pooled testing system with which to identify DRB3*009:02‐carrying cattle based on highly sensitive, specific real‐time PCR with TaqMan minor groove binder (MGB) probe design (DRB3*009:02‐TaqMan assay).

## 2 MATERIALS AND METHODS

### 2.1 Samples used for evaluating the diagnostic sensitivity and specificity of the DRB3*009: *02‐*TaqMan assay

Genomic DNA samples, which we extracted from the whole blood samples of 150 cattle in Japan, comprised 25 samples from a farm in Hokkaido prefecture, 116 from five farms in Miyazaki prefecture, and nine from a farm in which the presence of cattle carrying DRB3*009:02 had already been confirmed in Oita prefecture.^24^ Genomic DNA was extracted from each whole peripheral blood sample using the Wizard® Genomic DNA Purification Kit (Promega Corp., Madison, WI, USA) or the magLEAD Consumable Kit with an automated nucleic acid extraction system (magLEAD 12gC; Precision System Science Co., Ltd., Chiba, Japan) in accordance with the manufacturer's instructions.

To determine the DRB3*009:02‐TaqMan assay's performance for alleles that were not present in the current field samples, we used an artificially synthesized plasmid DNA containing the sequences of these alleles. For DRB3*009:01, the pcDNA3.1D/V5‐His‐TOPO® vector (Invitrogen, Thermo Fisher Scientific Inc., MA, USA), which contains the cattle DRB3*009:01 (ACNO: MT890683) cDNA that was constructed previously, was used. This plasmid DNA contained the sequence of each allele including the primer and probe sites designed in this study.

### 2.2 Primer–probe design

We designed primers and probe‐based selective assays for DRB3*009:02 (ACNO: LR797970). For the primer‐based selection, HiDi Taq DNA polymerase (myPOLS Biotec Konstanz, Germany), which has a discrimination ability at the 3′ terminal nucleotide of the primer, was used. For the probe‐based selection, the assay was designed to obtain a high‐melting temperature (Tm) value to enhance the MGB probe's specificity. Allele‐specific primers and probe were designed based on the 357 *DRB3* sequences obtained from the IPD‐MHC (major histocompatibility) database.^35^ These FASTA format sequences were first imported into MEGA X software,^36^ and sequence alignments were performed using ClustalW.^37^ On the basis of such alignments, minor nucleotides in DRB3*009:02 were chosen as candidates for the 3′ termini of the forward and reverse primer sites. We checked the internal sequences of the primer sets to identify the most suitable sequence with which to obtain a high‐specificity probe. Finally, the primer sequence was obtained by keeping the 3′ terminal nucleotide and adjusting the Tm. The primers and probe were custom‐made by Eurofins Genomics (Eurofins Genomics K.K., Tokyo, Japan).

### 2.3 Using conventional PCR to check the specificity of the primers

To check the specificity of the designed primer set, conventional PCR (cPCR) was performed with HiDi 2x PCR Master Mix (myPOLS Biotec). This reagent uses HiDi DNA polymerase, which has the same activity as HiDi Taq DNA polymerase. Genomic DNA from cattle heterozygous for DRB3*009:02 and 015:01, DRB3*034:01 and 005:03, or DRB3*001:01 and 014:01:01, and DRB3*009:01‐encoding plasmid DNA, were tested. We tested DRB3*009:02 and 009:01 because the complete sequences of the forward and reverse primers are identical and both will therefore be amplified. DRB3*034:01 was tested to check the discriminative ability of the primers because the forward primer's sequence is identical to DRB3*009:02, and only the 3′ terminal of the reverse primer's nucleotide differs from DRB3*009:02. DNA from cattle carrying DRB3*001:01 and 014:01:01 was tested to check for nonspecific reactions because these alleles do not have primers targeting their 3′ terminal nucleotides. The components of the reaction mix were set in accordance with the manufacturer's instructions from the HiDi 2x PCR Master Mix as well as the amplification profile.

### 2.4 DRB3*009: *02‐*TaqMan assay

PCRs were optimized in reaction mixes containing 0.3 μl of HiDi Taq DNA polymerase, 2.5 μl of 10 × HiDi reaction buffer, 200 μM of dNTPs (TOYOBO Co., Ltd., Osaka, Japan), 0.6 μM of each primer, 0.3 μM of probe, 0.1 μl of 50x Rox reference (Invitrogen, Thermo Fisher Scientific Inc.), 50 ng of template DNA, and PCR‐grade water up to 25 μl. The detailed quantity of each reagent used in the reaction mix is shown in Table S1. The conditions used for genome amplification involved an initial denaturation at 95°C for 2 min, 40 cycles of denaturation at 95°C for 10 s, and annealing and elongation at 65°C for 1 min. Real‐time PCRs were conducted using the QuantStudio 3 system (Applied Biosystems, Thermo Fisher Scientific Inc.). Samples were considered to be positive when Ct values of 0.15 ΔRn (an indicator of fluorescence) were obtained with fewer than 40 cycles and a positive signal.

To check the sensitivity and specificity of the DRB3*009:02‐TaqMan assay, genomic DNA from DRB3*009:02 and 015:01 heterozygous cattle and the plasmid DNA encoding the DRB3*009:01 sequence were tested. DRB3*009:01 was tested to check the probe's specificity because three nucleotides in the probe differ from DRB3*009:02 and both primer sequences were identical.

### 2.5 Identification of *DRB3* exon 2 alleles in field samples using PCR‐RFLP and sequencing‐based genotyping

We used PCR‐RFLP and DNA sequencing to identify *DRB3* exon 2 *(DRB3.2*) alleles in 150 field samples. The PCR‐PFLP method is frequently used for *DRB3.2* because it provides information on candidate alleles in samples; it can also distinguish heterozygous and homozygous alleles.^24^, ^29^, ^38^ The resultant PCR products were digested with *Rs*aI, *Hae*III, and *Bst*YI (New England Biolabs, MA, USA). First‐round *DRB3.2* PCRs were performed in 20 μl volumes each containing 0.2 μl of TaKaRa Ex Taq HS (TaKaRa Bio Inc., Shiga, Japan), 2 μl of 10x Ex Taq buffer (TaKaRa Bio Inc.), 1.6 μl of dNTP Mix (TaKaRa Bio Inc.), 0.2 μl of HL030 (5′‐ATCCTCTCTCTGCAGCACATTTC‐3′) and HL031 (5′‐TTTAAATTCGCGCTCACCTCGCCGCT‐3′) primers (10 μM each), 14.8 μl of PCR‐grade water, and 1 μl of template DNA. Second‐round PCRs were performed to increase the PCR product yield and obtain higher specificity. The 40 μl volumes each contained 0.2 μl of TaKaRa Ex Taq, 4 μl of 10x Ex Taq buffer, 3.2 μl of dNTP Mix, 0.2 μl of HL030 and HL032 primers (5′‐TCGCCGCTGCACAGTGAAACTCTC‐3′) (10 μM each), 30.2 μl of PCR‐grade water and 1 μl of the amplicon from the first‐round PCR. PCRs were performed using an initial denaturation of 98°C for 30 s, followed by 10 cycles (first‐round) or 35 cycles (second‐round) of denaturation at 98°C for 10 s, annealing at 60°C for 15 s, elongation at 72°C for 30 s, and a final extension at 72°C for 7 min. Second‐round amplicons (10 μl) were incubated with *Rsa*I and *Hae*III at 37°C for 6 h in 15 μl volumes containing 0.5 μl of enzyme (equal to 5 U), 1.5 μl of CutSmart Buffer (New England Biolabs), and 3 μl of PCR‐grade water. For the *Bst*YI reaction, each second‐round PCR amplicon was incubated with *Bst*YI at 60°C for 5 h in 15 μl volumes containing 0.5 μl of *Bst*YI (equal to 5 U), 1.5 μl of NEBuffer 2.1 (New England Biolabs), 10 μl of the second‐round PCR amplicon, and 3 μl of PCR‐grade water. The digested second‐round PCR amplicons were electrophoresed on 6% polyacrylamide gels to obtain their restriction patterns. Using this method, the restriction pattern numbers which represents *DRB3* alleles in same classification according to the combination of restriction pattern of these enzymes (defined in Reference 38), we identified were 1, 2, 3, 6, 7, 8, 10, 11, 15, 16, 18, 20, 21, 22, 23, 24, 25, 26, 27, 28, 32, 41, and 45. This indicates that the diversity of *DRB3.2* alleles in the samples used in this study were not biased toward specific alleles. Of note, samples that produced an E pattern with *Bst*YI indicate that the *DRB3.2* allele 11 is represented by either DRB3*009:01, 009:02, or 009:03. We identified 17 samples with this pattern.

Sanger sequencing was used for *DRB3.2* allele genotyping. The second‐round amplicons obtained from the PCR‐RFLP were extracted from 2% agarose gels using the QIAquick Gel Extraction Kit (QIAGEN, Hilden, Germany). HL030 and HL032 primers were used for sequencing with the Big Dye Terminator v3.1 cycle sequencing kit (Applied Biosystems, Thermo Fisher Scientific Inc.) and the Applied Biosystems 3130 Genetic Analyzer (Applied Biosystems, Thermo Fisher Scientific Inc.) in accordance with the manufacturer's instructions. The resulting data were analyzed using GENETYX Ver. 15 software (GENETYX Corp., Tokyo, Japan). Heterozygous base‐calling was used for the samples with heterozygous alleles. In such cases, *DRB3.2* alleles were determined by merging the sequence data and PCR‐RFLP patterns. For the 17 *DRB3.2* allele 11‐positive samples, we found that they were all were heterozygotes, including DRB3*009:02 (Table 1).

**TABLE 1:** Allele combinations for PCR‐RFLP heterozygous allele 11

ID	Allele 1	Allele 2
#1	*009:02*	*016:01*
#2	*009:02*	*015:01*
#3	*009:02*	*015:01*
#4	*009:02*	*002:01*
#5	*009:02*	*015:01*
#6	*009:02*	*007:01*
#7	*009:02*	*015:01*
#8	*009:02*	*015:01*
#9	*009:02*	*007:01*
#10	*009:02*	*007:01*
#11	*009:02*	*016:01*
#12	*009:02*	*016:01*
#13	*009:02*	*016:01*
#14	*009:02*	*016:01*
#15	*009:02*	*016:01*
#16	*009:02*	*010:01*
#17	*009:02*	*010:01*

### 2.6 Evaluating the diagnostic sensitivity and specificity of the DRB3*009: *02‐*TaqMan assay

Altogether, 150 field samples were used to evaluate the diagnostic sensitivity and specificity of the DRB3*009:02‐TaqMan assay compared with using combined PCR‐RFLP and sequencing analysis. The DRB3*009:02‐TaqMan assay was performed twice in independent reactions for all samples. Ct values at 0.15 ΔRn were obtained.

To determine the diagnostic sensitivity and specificity of the DRB3*009:02‐TaqMan assay with combined PCR‐RFLP–Sanger sequencing, the kappa value was calculated to measure the agreement between the two different methods; that is, whether the samples were positive or negative in the DRB3*009:02‐TaqMan assay versus DRB3*009:02 or not in the combined PCR‐RFLP–Sanger sequencing.

### 2.7 SYBR Green assay for DRB3*009:02

To compare the DRB3*009:02‐TaqMan assay's performance with the previously described SYBR Green assay,^39^ 150 field samples were tested using both methods. SYBR Green detection of DRB3*009:02 was performed in 20 μl reactions containing 10 μl of FastStart Universal SYBR Green Master (Rox) (Hoffmann‐La Roche Ltd., Basel, Switzerland), 0.6 μl of forward (5′‐CCTGGAGTATTCTAAGAGCG‐3′) and reverse (5′‐CGCCTCTCCTCCAGGATC‐3′) primers (10 μM each, equal to 0.3 μM), 6.8 μl of PCR‐grade water, and 2 μl of 25 ng/μl DNA (equal to 50 ng/reaction mixture). PCRs were performed using initial precycling (50°C for 2 min), initial denaturation (95°C for 10 min; 40 cycles of denaturation, 95°C for 10 s), and annealing and extension (60°C for 1 min). Post‐amplification melting curve analysis was performed. The real‐time PCRs were conducted using the QuantStudio 3 system. Ct values at 0.2 ΔRn were obtained. All samples were tested twice independently.

### 2.8 Application of DRB3*009: *02‐*TaqMan assay to the pooled testing system

The DRB3*009:02‐TaqMan assay's performance on pooled DNA samples was determined to further identify DRB3*009:02‐carrying cattle in a population with a low percentage of this allele. One DRB3*009:02‐positive sample was pooled with 29 DRB3*009:02–negative samples (1:29 pooling ratio) (DRB3*009:02‐containing DNA pool). This sample was used as a template for the DRB3*009:02‐TaqMan assay in the PCRs at 500, 250, 100, 50, 10, and 1 ng. Of note, 500, 250, 100, 50, 10, and 1 ng of the DRB3*009:02‐containing DNA pool contained 16.7, 8.3, 3.3, 1.67, 333, and 33.3 pg of DNA with DRB3*009:02, respectively. A DNA pool of 29 samples without DRB3*009:02 (DRB3*009:02‐NOT‐containing DNA pool) was also tested concurrently as the negative control. The PCR‐RFLP patterns of the 30 samples used in this test are shown in Table S2.

### 2.9 Regional survey of the percentage of DRB3*009:02‐carrying cattle in Kyushu Island, Japan

To determine the percentage of DRB3*009:02‐carrying cattle on Kyushu island, we employed the pooled testing system and individual testing based on the DRB3*009:02‐TaqMan assay. Altogether, 180 cattle blood samples from 115 farms in Kyushu Island were collected in ethylenediaminetetraacetic acid tubes from July to August 2021. The age of 173 of these cattle was 6–10 months. The others were more than 15 months old. All samples were stored at 4°C in the laboratory of the University of Miyazaki until testing.

Next, batches containing 30 individual blood samples were mixed together in Eppendorf tubes. Genomic DNAs from these pooled bloods were extracted using the Wizard® Genomic DNA Purification Kit or the magLEAD Consumable Kit with an automated nucleic acid extraction system in accordance with the manufacturer's instructions. The DRB3*009:02‐TaqMan assay identified the presence of the DRB3*009:02 allele in each pool. Finally, genomic DNA from all 180 blood samples was extracted using the methods described above and the DRB3*009:02‐TaqMan assay was performed individually on the samples. We calculated the percentage of cattle carrying DRB3*009:02 alleles by dividing the number of PCR‐positive individuals by the total number of samples × 100.

## 3 RESULTS

### 3.1 Primer/probe sequences and the performance of the DRB3*009: *02‐*TaqMan assay

**FIGURE 1:**
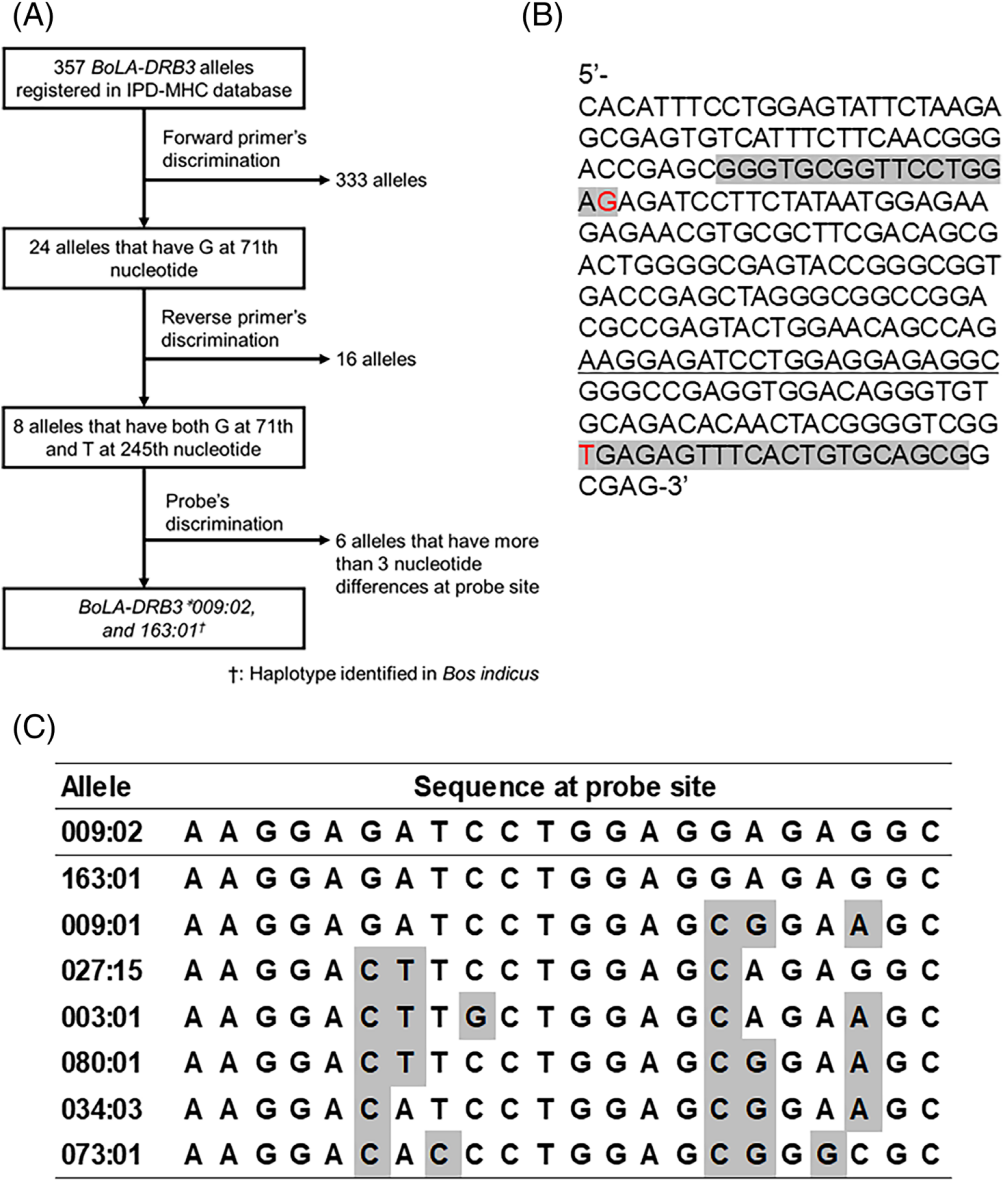
Primer and probe design for discriminating DRB3*009:02 from other alleles. (A) Schematic diagram showing the discrimination strategy used for DRB3*009:02. The efficiency of HiDi Taq DNA polymerase decreased dramatically when the 3′ terminal nucleotides in the primers and sample DNA differed. We therefore selected the 71st G for the target 3′ terminal nucleotide in the forward primer and 245th T for the target 3′ terminal nucleotide in the reverse primer. From 357 alleles, eight alleles including DRB3*009:02 were amplifiable by this primer. By merging the probe's discrimination ability, only DRB3*009:02 and DRB3*163:01 generated positive signals. (B) The primer and probe positions in the DRB3*009:02 sequence are shown. Gray highlight indicates the primer sites and underlining indicates the probe sites. The 3′ terminal nucleotides in the primers are indicated in red. (C) Sequences in the probe's site for eight alleles that were amplified by the primers are shown. Gray highlight indicates the different nucleotides in DRB3*009:02

On the basis of the discrimination ability at the 3′ terminal nucleotide in the primer site by HiDi Taq DNA polymerase, the 71st G and the 245th T in the 270‐bp DRB3*009:02 nucleotide sequence were selected for the 3′ terminal nucleotides of the forward and reverse primers, respectively (Figure 1A). The forward primer (5′‐GGGTGCGGTTCCTGGAG‐3′), reverse primer (5′‐CGCTGCACAGTGAAACTCTCA‐3′) and probe (5′‐FAM‐AAGGAGATCCTGGAGGAGAGGC‐MGB‐Eclipse‐3′) were designed (Figure 1B). By aligning eight alleles that reacted to restriction digests with the forward and reverse primers, we confirmed that the probe's specificity sufficiently discriminated DRB3*009:02 from other alleles, except DRB3*163:01 (Figure 1C).

**FIGURE 2:**
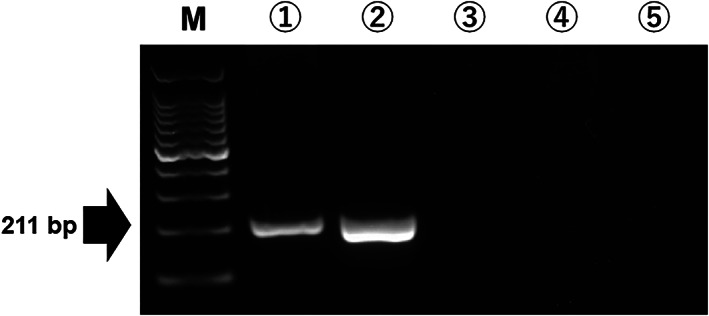
Performance of the designed primer set in cPCR. Electrophoresis results from cPCR using the designed primer set. 1: Genomic DNA from cattle heterozygous for DRB3*009:02 and 015:01. 2: Plasmid DNA encoding DRB3*009:01. 3: Genomic DNA from cattle heterozygous for DRB3*034:01 and 005:03. 4: Genomic DNA from cattle heterozygous for DRB3*001:01 and 14:01:01. 5: Negative control (PCR‐grade water)

As shown in Figure 2, the designed primer set only amplified genomic DNA from cattle that were heterozygous for DRB3*009:02 and 015:01 or the plasmid DNA encoding DRB3*009:01 in the cPCRs. As expected, genomic DNA from cattle that were heterozygous for DRB3*034:01 and 005:03 and heterozygous for DRB3*001:01 and 014:01:01 failed to amplify. Thus, we used the designed primer set for the DRB3*009:02‐TaqMan assay.

**FIGURE 3:**
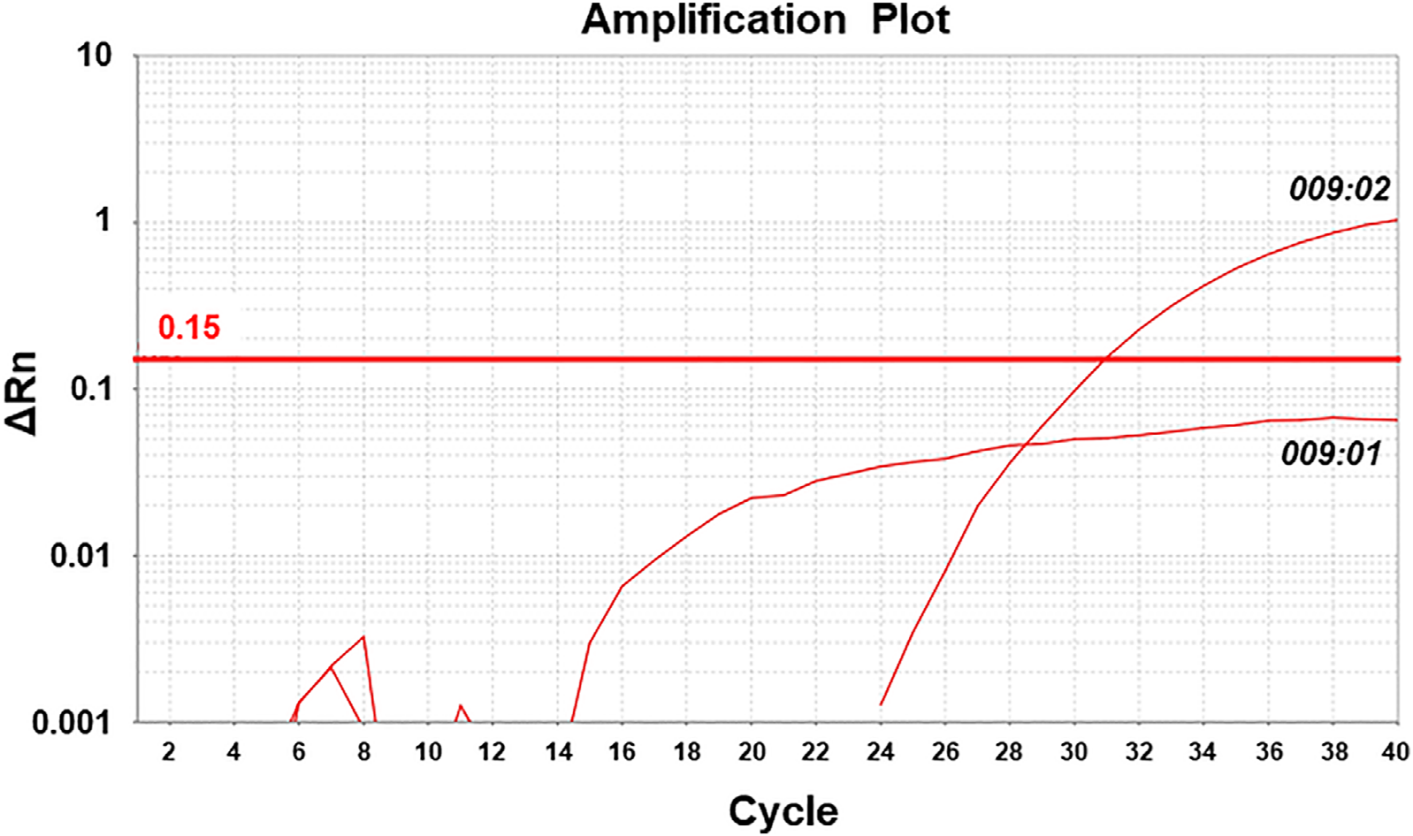
Discriminating DRB3*009:02 from 009:01 using the DRB3*009:02‐TaqMan assay. Amplification plot for the 50 ng samples of genomic DNA from cattle heterozygous for DRB3*009:02 and 015:01 and for 100 ng of plasmid DNA encoding the DRB3*009:01 sequence. Only genomic DNA from the cattle heterozygous for DRB3*009:02 and 015:01 produced positive signals

With the DRB3*009:01‐encoding plasmid DNA, the DRB3*009:02‐TaqMan assay produced negative signals in quantities varying from 1 fg to 100 ng, indicating a limited hybridization of the DRB3*009:02‐specific probe to DRB3*009:01. The amplification plot based on 50 ng genomic DNA samples from cattle that were heterozygous for DRB3*009:02 and *015:01* and that from 100 ng of plasmid DNA encoding the DRB3*009:01 sequence is shown in Figure 3.

### 3.2 Diagnostic sensitivity and specificity of the DRB3*009: *02‐*TaqMan assay

**FIGURE 4:**
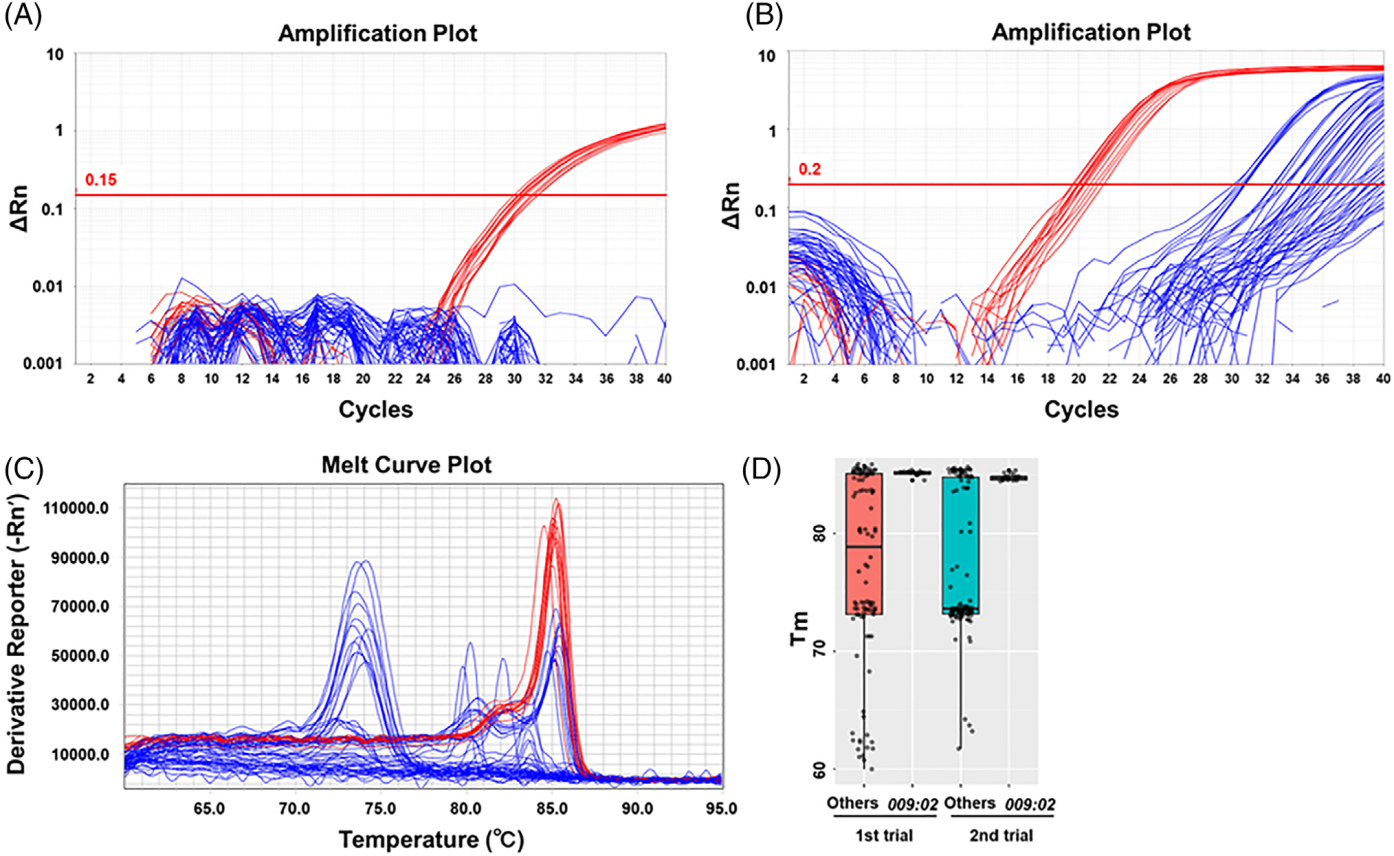
Performance of the DRB3*009:02‐TaqMan assay versus the SYBR Green assay. Amplification plots for 13 DRB3*009:02‐positive samples and 51 other samples. (A) DRB3*009:02‐TaqMan assay. (B) SYBR Green assay. (C) Post‐PCR melting curve for the samples obtained after PCR of (B). Red and blue lines in (A)–(C) indicate samples with DRB3*009:02 and other alleles, respectively. (D) Box and scatter plot of the Tm values from the melting peaks from the SYBR Green assay for 17 DRB3*009:02‐positive samples versus 133 others. The Y‐axis indicates the Tm and the gray points indicate each sample. Red and blue bars indicate the first and second trials, respectively. The black bars in the boxes indicate the median Tm values

As shown in Table 2 the results from the DRB3*009:02‐TaqMan assay versus PCR‐RFLP–Sanger sequencing completely matched; hence, the kappa value was 1. From these 150 field samples, 17 showed DRB3*009:02 positivity by producing positive signals (mean Ct value: 31.34; standard error, SE: ±0.20) in the DRB3*009:02‐TaqMan assay, whereas the others did not. Figure 4A shows the amplification plot from 13 of the DRB3*009:02‐positive samples and 51 other samples.

**TABLE 2:** Comparison between DRB3*009:02‐TaqMan assay versus combined PCR‐RFLP–Sanger sequencing

		PCR‐RFLP and sequencing	
		DRB3*009:02	Others
DRB3*009:02‐TaqMan assay	Positive	17	0
	Negative	0	133

### 3.3 Comparing the DRB3*009: *02‐*TaqMan assay with the SYBR Green assay

As shown in Figure 4B, the fluorescence signals from DRB3*009:02 in samples occurred early on in the SYBR Green assay. The mean Ct value in the 17 samples containing DRB3*009:02 was 21.12 (SE: ±0.32), whereas when DRB3*009:02 was absent in the DNA samples the mean Ct value was 35.06 (SE: ±0.18). All the DRB3*009:02‐lacking DNA samples generated fluorescence signals exceeding Ct values of 29.50, or no signals at all.

A comparison of the Ct values from the 17 DRB3*009:02‐positive samples in the two trials between the DRB3*009:02‐TaqMan assay and the SYBR Green assay is shown in Table 3. One sample (sample #10) had a Ct value of 30.58 in the second test in the SYBR Green assay. When checking the Tm values for the melting peaks, we noticed that some DNA samples that did not contain DRB3*009:02 had similar Tm values as those from the DRB3*009:02‐positive DNA samples (Figure 4C,D). Thus, we were unable to differentiate DNA samples containing DRB3*009:02 from those with false negative signals and others using the Tm values of the melting peaks. However, the DRB3*009:02‐TaqMan assay showed superior Ct reproducibility in both trials (Table 3).

**TABLE 3:** Ct values for genomic DNA samples from BoLA‐DRB3*009:02‐carrying cattle from DRB3*009:02‐TaqMan and SYBR Green assays

	DRB3*009:02‐TaqMan assay		SYBR Green assay			
ID	Ct in 1st trial	Ct in 2nd trial	Ct in 1st trial	Tm in 1st trial	Ct in 2nd trial	Tm in 2nd trial
#1	35.09	34.97	22.00	85.16	22.06	84.97
#2	32.76	32.03	19.78	85.27	19.66	85.17
#3	31.57	31.82	20.73	85.16	20.70	85.17
#4	31.97	31.71	21.10	85.14	21.14	85.36
#5	31.26	31.71	21.39	84.52	21.34	84.43
#6	30.25	30.54	20.13	85.02	20.72	84.68
#7	30.50	30.36	20.51	85.24	20.88	84.74
#8	31.09	31.65	20.43	85.31	22.48	84.76
#9	31.54	31.59	20.33	85.41	22.47	84.71
#10	31.26	30.73	19.61	85.26	30.58^a^	84.86^a^
#11	30.60	31.05	19.73	85.26	20.35	84.86
#12	30.20	30.72	20.17	85.21	20.81	84.76
#13	30.76	31.23	21.70	85.04	21.89	84.50
#14	29.95	30.31	19.94	85.20	21.64	84.55
#15	30.32	30.45	21.15	85.04	20.21	84.55
#16	30.56	30.55	20.00	84.99	20.07	84.55
#17	32.37	32.21	20.80	84.99	21.56	84.50

^a^ Indicates a sample that contains DRB3*009:02 but had a false negative signal.

### 3.4 Performance of the pooled testing system based on the DRB3*009: *02‐*TaqMan assay

**FIGURE 5:**
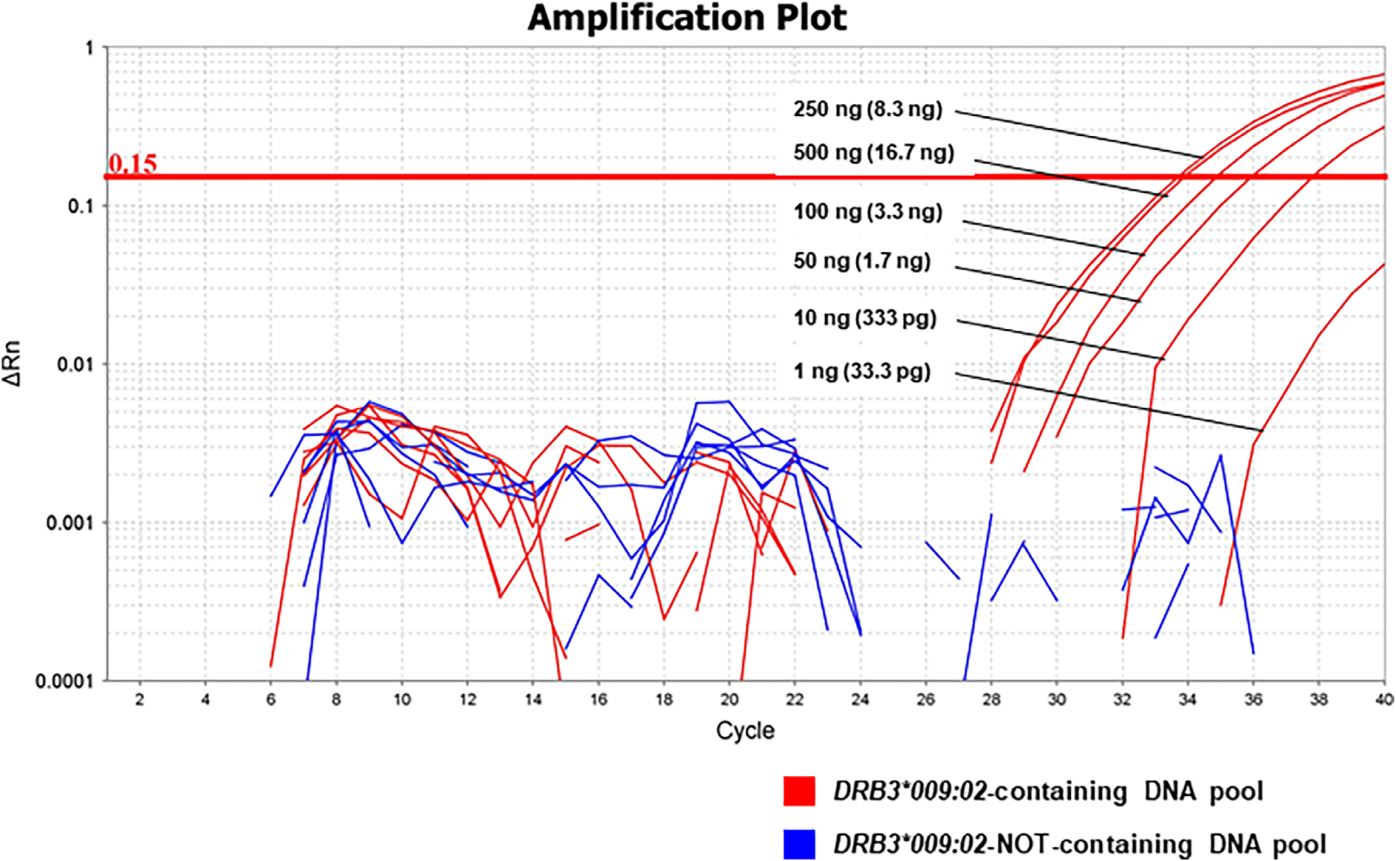
Performance of the DRB3*009:02‐TaqMan assay at detecting DRB3*009:02‐carrying cattle DNA from pooled DNA. Amplification plots for the 500, 250, 100, 50, 10, and 1 ng of DRB3*009:02‐containing DNA pool and DRB3*009:02‐NOT‐containing DNA pool are shown. Red and blue lines indicate the DRB3*009:02‐containing DNA pool and DRB3*009:02‐NOT‐containing DNA pool, respectively. The blank area underneath the DNA amounts in the reaction mixtures indicates the amount of DNA used for detecting DRB3*009:02. Ct values above 0.15 ΔRn in the DRB3*009:02‐containing DNA pool were 33.85 (500 ng), 33.66 (250 ng), 34.82 (100 ng), 35.91 (50 ng), and 37.74 (10 ng); 1 ng = not detected

The performance of the DRB3*009:02‐TaqMan assay on pooled samples was determined using pooled DNAs. One DNA pool contained DNAs from one animal carrying DRB3*009:02 and 29 other cattle without DRB3*009:02 (the DRB3*009:02‐containing DNA pool). Another DNA pool contained DNAs from cattle without DRB3*009:02 (the DRB3*009:02‐NOT‐containing DNA pool). As shown in Figure 5, the DRB3*009:02‐TaqMan assay produced positive signals in the 500, 250, 100, 50, and 10 ng DRB3*009:02‐containing DNA pool, but failed to detect DRB3*009:02 with 1 ng of DNA in this pool. This indicates that the DRB3*009:02‐TaqMan assay can detect DRB3*009:02 in DNA in a pooled sample when at least 333 pg of it is contained in the reaction mixture. Furthermore, none of the DNA quantities (1 ng–500 ng) in the DRB3*009:02‐NOT‐containing DNA pool produced positive signals, indicating that no nonspecific amplification occurred in the tested pooled samples.

### 3.5 Percentage of cattle carrying DRB3*009:02 in Kyushu Island, Japan

Six pools from 180 samples were tested. As shown in Table 4, all the pools produced positive signals. Individual testing revealed that 19 samples were DRB3*009:02 allele‐positive and that all the pools contained blood from cattle carrying DRB3*009:02 (Table 4). The DRB3*009:02 status of each sample is shown in Table S3. The percentage of cattle carrying DRB3*009:02 in the Kyushu Island was 10.56% (19/180 cattle, 95% confidence interval: 6.64%–16.22%).

**TABLE 4:** Detection of cattle carrying DRB3*009:02 using the pooled testing system and individual testing

Pool No.	No. of pooled samples	Pooled testing results	No. of positive animals in pooled samples
1	30	Positive	2
2	30	Positive	3
3	30	Positive	3
4	30	Positive	5
5	30	Positive	1
6	30	Positive	5

## 4 DISCUSSION

Cattle carrying the DRB3*009:02 allele are strongly associated with BLV dissemination resistance, as shown by having undetectable provirus levels and a lack of disease progression when infected with BLV, compared with other cattle.^20^, ^21^, ^22^, ^23^, ^24^ By considering the presence of minor population of cattle carrying DRB3*009:02, which have detectable BLV provirus,^25^, ^26^, ^27^, ^28^ property of this allele should be used for the screening of BLV ECs. BLV ECs can be characterized by satisfaction of both this allele and undetectable provirus. Using BLV ECs to control BLV infection will necessitate genotyping cattle for the presence of this allele. However, the percentage of cattle in the population carrying the DRB3*009:02 allele is low.^24^ To identify cattle with DRB3*009:02 alleles in a large population rapidly, we found that employing a pooled testing system based on the DRB3*009:02‐TaqMan assay was useful for screening in terms of time and cost.

Currently, 357 *DRB3* alleles are registered in the IPD‐MHC database. *DRB3* is one of the most polymorphic regions in the bovine MHC and many similar sequences exist. Our DRB3*009:02‐TaqMan assay detected DRB3*009:02 in field samples with high accuracy and reproducibility, as compared with the previously described SYBR Green assay.^39^ The specificity of the TaqMan MGB probe combined with primer discrimination based on HiDi Taq polymerase was sufficient for *DRB3* genotyping because of its high specificity. This method is applicable in medicine to identify individuals with genetic resistance against other pathogens, such as the ECs of HIV, with more specificity.

Two other methods have been used to identify cattle carrying DRB3*009:02: sequence‐based typing (SBT)^40^, ^41^, ^42^ and PCR‐RFLP.^24^, ^29^, ^38^ SBT combines PCR with allele specific‐primers and uses a computer‐controlled algorism to interpret the Sanger sequencing results. Although this method enables high‐throughput *DRB3* genotyping, the problems of uncorrected genotyping results from heterozygous base‐calling remain. This method also depends on the quality of the Sanger sequencing base‐calling. In contrast, the PCR‐RFLP method is a platform for *DRB3* genotyping in that it uses the restriction fragment patterns from the PCR amplicons. However, this method is unable to distinguish alleles with the same restriction pattern. Our team previously observed three cattle with DRB3*009:01 and 57 with DRB3*009:02 alleles among 60 cattle with the E pattern of *Bst*YI restriction.^24^ Therefore, SBT and PCR‐RFLP are hampered by specificity. Unlike them, by offering specificity benefit, the DRB3*009:02‐TaqMan assay is a practical diagnostic genotyping tool satisfying its requirement.

BLV infections have reportedly occurred in more than 56 countries.^14^ In the current epidemic situation, completely eradicating BLV from the field by culling infected cattle is not economically feasible. Appropriate BLV control that involves keeping BLV‐infected cattle is needed. One solution is to isolate BLV‐infected cattle from the main herd or barn.^15^ This control strategy, however, requires enough space and is therefore limited by the capacity of the farm. To heighten BLV control under this situation, we recommended that BLV ECs are used as a barrier against BLV transmission by positioning them between infected cattle herds and uninfected ones. Thus, sufficient numbers of BLV ECs should be secured. We determined that the percentage of DRB3*009:02‐carrying cattle in Kyushu Island was 10.56% (19/180 cattle, 95% confidence interval: 6.64%–16.22%) (Table 4). This result is consistent with our previous study indicating that cattle carrying DRB3*009:02 existed in 6.8% of the cattle population in the same region.^24^ Cattle carrying DRB3*009:02 have been seen in Japan and across the world.^43^, ^44^, ^45^, ^46^, ^47^, ^48^, ^49^, ^50^ On the other hands, not all cattle carrying DRB3*009:02 can be BLV ECs. Previous reports showed that minor population of cattle carrying this allele had detectable BLV provirus and progressed lymphoma.^25^, ^26^, ^27^, ^28^ This is most likely because BLV resistance is determined by not only a single *DRB3* allele but also combinations of *DRB3* heterozygous allele and/or other factors, even when DRB3*009:02 seems to strongly affect BLV dissemination resistance.^29^, ^30^ Furthermore, cattle carrying DRB3*009:02 have detectable provirus in the early stage of BLV infection.^51^ To avoid using cattle carrying DRB3*009:02 with detectable BLV provirus, we recommend characterizing BLV ECs by DRB3*009:02 and absence of BLV provirus. BLV ECs maintain status of undetectable BLV provirus in a long span.^52^ We expect that identifying BLV ECs and the challenge of global BLV control should be made easier by using a pooled testing system based on the DRB3*009:02‐TaqMan assay.

The breeding strategy employed for cattle is also a key aspect of disease control. After identifying DRB3*009:02‐carrying cattle, maintaining this allele in the cattle continuously will be needed. Thus, selective breeding based on the genetic information of the parents will be required. The appropriate use of genetic screening or engineering in food animals would help with the food safety of cattle products, one example of which is the production of prion gene‐knockout cattle.^53^ However, maintaining genetic species diversity is also important for retaining the various characteristics associated with disease resistance. Hence, producing cattle carrying DRB3*009:02 alleles by selective breeding while keeping cattle with other *DRB3* alleles is a good option.

This study has one limitation. The DRB3*009:02‐TaqMan assay cannot discriminate DRB3*163:01 among the alleles registered in the IPD‐MHC database. DRB3*163:01 was identified in *Bos indicus*. Therefore, the DRB3*009:02‐TaqMan assay is recommended as a screening test for DRB3*009:02 when testing *B. indicus*. Of note, the SYBR Green assay also has difficulty differentiating DRB3*163:01 from DRB3*009:02, but PCR‐RFLP can discriminate these alleles. The sequence homology between DRB3*163:01 and DRB3*009:02 is 98.1%, and the loci of the different nucleotides within DRB3*009:02 are sporadic. We are interested in determining the susceptibility to BLV of cattle carrying the former allele.

## 5 CONCLUSIONS

A pooled testing system is available for easy screening of cattle carrying DRB3*009:02 at herd‐level with low cost and without excessive labor. The DRB3*009:02‐TaqMan assay shows high‐discriminative sensitivity and specificity toward DRB3*009:02, making it suitable for identifying cattle carrying DRB3*009:02 in individual post‐screening tests. This reliable diagnostic laboratory tool is applicable in selective breeding. This strategy should contribute to BLV control through the use of BLV ECs.

## CONFLICT OF INTEREST

The authors declare that they have no conflict of interest.

## AUTHOR CONTRIBUTIONS

Kosuke Notsu contributed to funding acquisition, data collection, and analysis, manuscript writing. Hala El Daous contributed to data collection, analysis, and manuscript review. Shuya Mitoma contributed to analysis and manuscript review. Junzo Norimine reviewed the manuscript. Satoshi Sekiguchi contributed to conceptualization, funding acquisition, and supervision. All authors have read and approved the final manuscript.

## Supporting information


**Table S1** Composition of the reaction mixture used for the DRB3*009:02‐TaqMan assay.

**Table S2** List of the PCR‐RFLP patterns of each sample used to evaluate the performance of the DRB3*009:02‐TaqMan assay on pooled DNA.

**Table S3** Description of each sample used to survey the percentage of DRB3*009:02‐carrying cattle on Kyushu Island, Japan.

## Data Availability

All the data generated or analyzed in this study are included in the published article and its additional files.
