# Supplementary material for: A pooled testing system to rapidly identify cattle carrying the elite controller BoLA‐DRB3*009:02 haplotype against bovine leukemia virus infection
Source: HLA. 2021 Dec 19;99(1):12–24. doi: 10.1111/tan.14502 (PMC9543338; doi:10.1111/tan.14502)
Supplement: Supplementary file 1 — Table S1 Composition of the reaction mixture used for the DRB3*009:02‐TaqMan assay. [file TAN-99-12-s002.docx]

| **Table S1. Composition of the reaction mixture used for the *DRB3*009:02-*TaqMan assay** | |
| --- | --- |
| **Components** | **×1 (μL)** |
| **5 U/µl-HiDi Taq polymerase** | **0.3** |
| **10× HiDi reaction buffer** | **2.5** |
| **2 mM dNTP mixture** | **2.5** |
| **10 μM-Forward primer** | **1.5** |
| **10 μM-Reverse primer** | **1.5** |
| **10 μM-Probe** | **0.75** |
| **50×ROX Reference** | **0.1** |
| **PCR-grade water** | **13.85** |
| **25 ng/μL Template DNA** | **2** |
| **Total** | **25** |
