# Supplementary material for: A pooled testing system to rapidly identify cattle carrying the elite controller BoLA‐DRB3*009:02 haplotype against bovine leukemia virus infection
Source: HLA. 2021 Dec 19;99(1):12–24. doi: 10.1111/tan.14502 (PMC9543338; doi:10.1111/tan.14502)
Supplement: Supplementary file 2 — Table S2 List of the PCR‐RFLP patterns of each sample used to evaluate the performance of the DRB3*009:02‐TaqMan assay on pooled DNA. [file TAN-99-12-s001.docx]

| **Table S2. List of the PCR-RFLP patterns of each sample used to evaluate the performance of the *DRB3*009:02*-TaqMan assay on pooled DNA** | |
| --- | --- |
| Sample No. | PCR-RFLP Pattern |
| #1 | 2/3 |
| #2 | 15/15 |
| #3 | 16/16 |
| #4 | 16/16 |
| #5 | 16/2 |
| #6 | 27/6 |
| #7 | 10/10 |
| #8 | 10/10 |
| #9 | 7/10 |
| #10 | 10/8 |
| #11 | 10/10 |
| #12 | 15/28 |
| #13 | 10/3 |
| #14 | 3/10 |
| #15 | 1/45 |
| #16 | 16/10 |
| #17 | 23/23 |
| #18 | 23/24 |
| #19 | 16/3 |
| #20 | 28/3 |
| #21 | 27/22 |
| #22 | 23/24 |
| #23 | 24/7 |
| #24 | 7/7 |
| #25 | 8/10 |
| #26 | 23/24 |
| #27 | 27/15 |
| #28 | 22/27 |
| #29 | 24/24 |
| #30 | 11/ 28 (*DRB3*009:02* and *007:01*) |
