# Supplementary material for: A pooled testing system to rapidly identify cattle carrying the elite controller BoLA‐DRB3*009:02 haplotype against bovine leukemia virus infection
Source: HLA. 2021 Dec 19;99(1):12–24. doi: 10.1111/tan.14502 (PMC9543338; doi:10.1111/tan.14502)
Supplement: Supplementary file 3 — Table S3 Description of each sample used to survey the percentage of DRB3*009:02‐carrying cattle on Kyushu Island, Japan. [file TAN-99-12-s003.docx]

| **Table S3. Description of each sample used to survey the percentage of *DRB3*009:02*-carrying cattle on Kyushu Island, Japan** | | | |
| --- | --- | --- | --- |
| Sample No. | Pool No. | Pooled testing results | Individual testing results |
| #1 | 1 | + | - |
| #2 |  |  | - |
| #3 |  |  | - |
| #4 |  |  | - |
| #5 |  |  | - |
| #6 |  |  | - |
| #7 |  |  | - |
| #8 |  |  | - |
| #9 |  |  | - |
| #10 |  |  | - |
| #11 |  |  | - |
| #12 |  |  | - |
| #13 |  |  | - |
| #14 |  |  | - |
| #15 |  |  | - |
| #16 |  |  | - |
| #17 |  |  | - |
| #18 |  |  | - |
| #19 |  |  | - |
| #20 |  |  | - |
| #21 |  |  | - |
| #22 |  |  | + |
| #23 |  |  | - |
| #24 |  |  | - |
| #25 |  |  | - |
| #26 |  |  | + |
| #27 |  |  | - |
| #28 |  |  | - |
| #29 |  |  | - |
| #30 |  |  | - |
| #31 | 2 | + | + |
| #32 |  |  | - |
| #33 |  |  | - |
| #34 |  |  | - |
| #35 |  |  | - |
| #36 |  |  | - |
| #37 |  |  | - |
| #38 |  |  | - |
| #39 |  |  | - |
| #40 |  |  | - |
| #41 |  |  | + |
| #42 |  |  | - |
| #43 |  |  | - |
| #44 |  |  | - |
| #45 |  |  | - |
| #46 |  |  | - |
| #47 |  |  | - |
| #48 |  |  | - |
| #49 |  |  | - |
| #50 |  |  | - |
| #51 |  |  | - |
| #52 |  |  | - |
| #53 |  |  | - |
| #54 |  |  | - |
| #55 |  |  | - |
| #56 |  |  | - |
| #57 |  |  | + |
| #58 |  |  | - |
| #59 |  |  | - |
| #60 |  |  | - |
| #61 | 3 | + | - |
| #62 |  |  | - |
| #63 |  |  | - |
| #64 |  |  | + |
| #65 |  |  | - |
| #66 |  |  | - |
| #67 |  |  | - |
| #68 |  |  | - |
| #69 |  |  | - |
| #70 |  |  | - |
| #71 |  |  | - |
| #72 |  |  | - |
| #73 |  |  | - |
| #74 |  |  | - |
| #75 |  |  | - |
| #76 |  |  | - |
| #77 |  |  | + |
| #78 |  |  | - |
| #79 |  |  | + |
| #80 |  |  | - |
| #81 |  |  | - |
| #82 |  |  | - |
| #83 |  |  | - |
| #84 |  |  | - |
| #85 |  |  | - |
| #86 |  |  | - |
| #87 |  |  | - |
| #88 |  |  | - |
| #89 |  |  | - |
| #90 |  |  | - |
| #91 | 4 | + | + |
| #92 |  |  | - |
| #93 |  |  | - |
| #94 |  |  | - |
| #95 |  |  | - |
| #96 |  |  | + |
| #97 |  |  | - |
| #98 |  |  | - |
| #99 |  |  | - |
| #100 |  |  | - |
| #101 |  |  | - |
| #102 |  |  | - |
| #103 |  |  | - |
| #104 |  |  | - |
| #105 |  |  | - |
| #106 |  |  | - |
| #107 |  |  | - |
| #108 |  |  | - |
| #109 |  |  | - |
| #110 |  |  | - |
| #111 |  |  | - |
| #112 |  |  | - |
| #113 |  |  | - |
| #114 |  |  | + |
| #115 |  |  | + |
| #116 |  |  | - |
| #117 |  |  | - |
| #118 |  |  | - |
| #119 |  |  | + |
| #120 |  |  | - |
| #121 | 5 | + | - |
| #122 |  |  | - |
| #123 |  |  | + |
| #124 |  |  | - |
| #125 |  |  | - |
| #126 |  |  | - |
| #127 |  |  | - |
| #128 |  |  | - |
| #129 |  |  | - |
| #130 |  |  | - |
| #131 |  |  | - |
| #132 |  |  | - |
| #133 |  |  | - |
| #134 |  |  | - |
| #135 |  |  | - |
| #136 |  |  | - |
| #137 |  |  | - |
| #138 |  |  | - |
| #139 |  |  | - |
| #140 |  |  | - |
| #141 |  |  | - |
| #142 |  |  | - |
| #143 |  |  | - |
| #144 |  |  | - |
| #145 |  |  | - |
| #146 |  |  | - |
| #147 |  |  | - |
| #148 |  |  | - |
| #149 |  |  | - |
| #150 |  |  | - |
| #151 | 6 | + | - |
| #152 |  |  | - |
| #153 |  |  | - |
| #154 |  |  | - |
| #155 |  |  | - |
| #156 |  |  | - |
| #157 |  |  | - |
| #158 |  |  | - |
| #159 |  |  | - |
| #160 |  |  | - |
| #161 |  |  | - |
| #162 |  |  | + |
| #163 |  |  | - |
| #164 |  |  | - |
| #165 |  |  | + |
| #166 |  |  | - |
| #167 |  |  | - |
| #168 |  |  | - |
| #169 |  |  | + |
| #170 |  |  | - |
| #171 |  |  | + |
| #172 |  |  | - |
| #173 |  |  | - |
| #174 |  |  | - |
| #175 |  |  | + |
| #176 |  |  | - |
| #177 |  |  | - |
| #178 |  |  | - |
| #179 |  |  | - |
| #180 |  |  | - |
|  |  |  | +: PCR positive |
|  |  |  | -: PCR negative |
